# Choline Acetyltransferase Induces the Functional Regeneration of the Salivary Gland in Aging SAMP1/Kl -/- Mice

**DOI:** 10.3390/ijms22010404

**Published:** 2021-01-02

**Authors:** Nguyen Khanh Toan, Nguyen Chi Tai, Soo-A Kim, Sang-Gun Ahn

**Affiliations:** 1 Department of Pathology, School of Dentistry, Chosun University, Gwangju 61452, Korea; nguyenkhanhtoant57@gmail.com (N.K.T.); taik58cnsh@gmail.com (N.C.T.); 2 Department of Biochemistry, School of Oriental Medicine, Dongguk University, Gyeongju 38066, Korea; ksooa@dongguk.ac.kr

**Keywords:** salivary gland dysfunction, SAMP1/Klotho, Choline acetyltransferase (ChAT), Acetylcholine

## Abstract

Salivary gland dysfunction induces salivary flow reduction and a dry mouth, and commonly involves oral dysfunction, tooth structure deterioration, and infection through reduced salivation. This study aimed to investigate the impact of aging on the salivary gland by a metabolomics approach in an extensive aging mouse model, SAMP1/Klotho -/- mice. We found that the salivary secretion of SAMP1/Klotho -/- mice was dramatically decreased compared with that of SAMP1/Klotho WT (+/+) mice. Metabolomics profiling analysis showed that the level of acetylcholine was significantly decreased in SAMP1/Klotho -/- mice, although the corresponding levels of acetylcholine precursors, acetyl-CoA and choline, increased. Interestingly, the mRNA and protein expression of choline acetyltransferase (ChAT), which is responsible for catalyzing acetylcholine synthesis, was significantly decreased in SAMP1/Klotho -/- mice. The overexpression of ChAT induced the expression of salivary gland functional markers (α–amylase, ZO-1, and Aqua5) in primary cultured salivary gland cells from SAMP1/Klotho +/+ and -/- mice. In an in vivo study, adeno-associated virus (AAV)-ChAT transduction significantly increased saliva secretion compared with the control in SAMP1/Klotho -/- mice. These results suggest that the dysfunction in acetylcholine biosynthesis induced by ChAT reduction may cause impaired salivary gland function

## 1. Introduction

Salivary gland dysfunction is caused by radiation, medications, autoimmune or metabolic diseases, and aging-related degeneration is a common etiology of salivary gland impairment. Aging leads to structural changes in the salivary gland via the replacement of acinar cells by fat and fibrous tissues in the submandibular, parotid, and minor glands [1,2]. Since saliva is primarily produced in acinar cells, salivary gland degeneration can lead to hyposalivation in elderly individuals [3,4]. Moreover, several studies have shown a correlation between aging and reduced components in saliva, including digestive enzymes, hormones, antimicrobial substances, growth factors, and antioxidants [5,6,7]. A decrease in saliva has severe effects on the quality of life of patients, as it deteriorates taste perception, swallowing, and speech performance [8]. Decreased saliva also leads to an increase in the incidence of dental caries, mucosal atrophy, and ulceration, in addition to an overall reduction in the immune response against infectious agents [9]. More recent research has suggested that saliva output in the major glands may diminish with increasing age and may occur with aging-related changes in salivary viscoelasticity in submandibular and sublingual glands [10]. In contrast to the above, some studies suggest that clinically significant declines in the stimulated saliva flow rate may not be significantly impacted by age [11]. Although there are conflicting results, a recent meta-analysis suggested that there is an age-related reduction in saliva secretion in resting and stimulated saliva in elderly adults compared with young adults [12]. In addition, population-based studies have demonstrated diminished salivary gland size, saliva volume, and saliva components with increasing age [9,13,14,15]. However, although aging has been reported to decrease salivary gland function, the fundamental mechanism is not clear.

In our previous study, we analyzed the gene expression profile in salivary glands from accelerated aging Klotho-deficient mice [16]. Recently, we demonstrated that activation of KLF4 signaling contributes to potentiating the function of salivary glands using aging Klotho mice [17]. Accelerated aging Klotho-deficient mouse models have displayed phenotypes in multiple organ systems that suggest premature aging and resemble features of natural aging of both mice and humans. Klotho-deficient mice were established as a model for extensively studying aging mechanisms because of their short lifespan and multiple aging-like phenotypes, including growth retardation, cognitive impairment, skin and muscle atrophy, vascular calcification, and osteoporosis [18]. In Klotho-deficient mice, the salivary glands contain eosinophilic granules and decreased granular ducts and lobes and nerve growth factor (NGF)- and epidermal growth factor (EGF)-positive ducts [19]. Additionally, various metabolic abnormalities are induced in Klotho-deficient mice, such as hypoglycemia, lack of glycogen storage, and low fat in brown adipose tissue [20]. We also demonstrated that the levels of metabolites associated with the central carbon, amino acid, and coenzyme pathways and with trimethylaminuria metabolism, glutathione (GSH) metabolism, the pentose–phosphate pathway, and nicotinate/nicotinamide metabolism differed in the kidneys of aging-accelerating SAMP1/kl -/- mice [21]. Therefore, we hypothesise that there might be a connection between aging-induced salivary gland dysfunction and metabolism disruption. We conducted this study to analyze the mechanism of aging-induced salivary gland dysfunction on the metabolomics approach.

In this study, we investigated metabolic alterations that may be related to salivary gland dysfunction in SAMP1/kl -/- mice. We found that aging SAMP1/kl -/- mice exhibit lower saliva volume and impaired acetylcholine (Ach) metabolism than SAMP1/kl+/+ mice. This alteration is linked with a decrease in the expression of choline acetyltransferase (ChAT) at both transcriptional and translational levels. ChAT overexpression restored the Ach concentration and improved the function of the salivary gland. Consistently, in vivo adeno-associated virus (AAV)-ChAT transduction improved the salivary gland function of SAMP1/kl -/- mice. The age-related alteration in the expression of ChAT in SAMP1/Kl -/- mice might account for the concomitant changes in salivary gland activity. These results suggest that the ChAT/Ach pathway might have therapeutic benefit for the treatment of age-related salivary gland dysfunction.

## 2. Results

### 2.1. Metabolic Profiling of Salivary Glands in SAMP1/kl +/+ and SAMP1 kl -/- Mice

**Figure 1:**
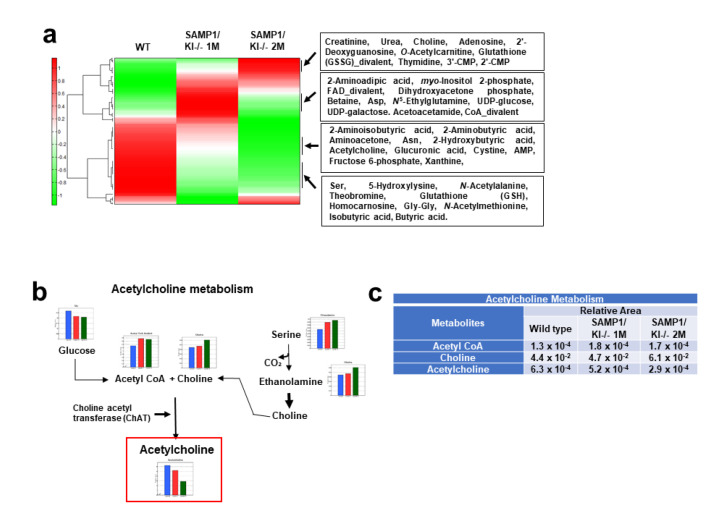
A comparison of the metabolic profile of mouse salivary gland tissues (SAMP1/kl +/+, 1-month-old SAMP1/kl -/-, and 2-month-old SAMP1/kl -/-) was conducted by capillary electrophoresis time-of-flight mass spectrometry (CE-TOFMS). (**a**) Hierarchical cluster analysis (HCA) and heatmap visualization of metabolic profiles. The horizontal axis and vertical axis show the sample names and peaks, respectively. HCA was performed for the peaks. The distance between peaks is displayed in tree diagrams. (**b**) The level of metabolites of acetylcholine metabolism was plotted on the pathway maps. The relative quantities of detected metabolites are represented as bar graphs (from left to right: SAMP1/kl +/+ (blue), 1-month-old SAMP1/kl -/- (red), and 2-month-old SAMP1/kl -/- (green) salivary glands). The red block highlighted the Acetylcholine concentration. (**c**) Comparisons of the relative amount of acetylcholine metabolites between SAMP1/kl +/+ salivary glands and SAMP1/kl -/- salivary glands at 1 month and 2 months.

Metabolome analysis was performed with mouse salivary gland tissue (SAMP1/kl +/+, 1-month-old SAMP1/kl -/-, and 2-month-old SAMP1/kl -/- animals) in two capillary electrophoresis time-of-flight mass spectrometry (CE-TOFMS) modes to identify cationic and anionic metabolites. We detected 232 metabolites (134 metabolites in cation mode and 98 metabolites in anion mode) based on the HMT standard library. The results for the 232 detected peaks are summarized in Supplementary Table S1. The heat map shows the comparison of the metabolic profiles (Figure 1a). In the heat map analysis, we observed that there was a clear difference between the SAMP1/kl+/+ and 1-month-old SAMP1/kl -/- or 2-month-old SAMP1/kl -/- samples (Figure 1a). The PCA analysis revealed that the difference between SAMP1/kl +/+ and 1-month-old SAMP1 kl -/- or 2-month-old SAMP1/kl -/- contributed 66.4% of the total variance, while the difference between 1-month-old and 2-month-old SAMP1 kl -/- contributed to only 33.7% of the total variance. The metabolic pathways associated with all the detected metabolites are illustrated in Supplementary Figure S1. In the metabolic pathway analysis, we found that there was a noticeable difference in acetylcholine (Ach) metabolites between SAMP1/kl +/+ and 1-month-old or 2-month-old SAMP1/kl -/-. Ach is the primary neurotransmitter in the parasympathetic nervous system involved in multiple processes, including the stimulation of saliva secretion. The amount of Ach was reduced significantly in SAMP1 kl -/- animals at 1 month and 2 months compared with SAMP1 kl +/+ animals, while the amount of Ach precursors—acetyl-CoA and choline—were not significantly decreased in SAMP1 kl -/- animals at 1 month or 2 months. The values of the metabolites involved in Ach metabolism is plotted in Figure 1b,c. As shown in Figure 1c, Ach levels were reduced by nearly 20% in SAMP1 kl -/- at 1 month and nearly 55% in SAMP1 kl -/- at 2 months compared to SAMP1 kl +/+. On the other hand, acetyl-CoA and choline levels were slightly increased in SAMP1 kl -/- at 1 month and SAMP1 kl -/- at 2 months. In general, the biosynthesis of Ach from acetyl-CoA and choline is mediated by choline acetyltransferase (ChAT). Therefore, these data suggested that the decrease in Ach in the SAMP1 kl -/- model may be linked with the attenuation of ChAT expression/activity

### 2.2. Saliva Secretion Mediated by Acetylcholine (Ach) in SAMP1/kl -/- Mice

**Figure 2:**
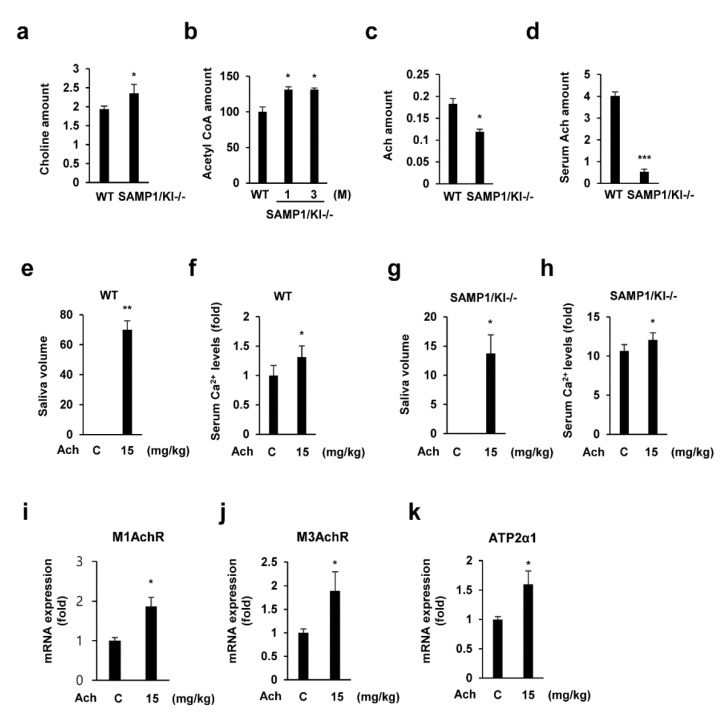
Age-related acetylcholine metabolism alteration and acetylcholine-induced modification in the SAMP1/kl model. (**a**–**d**) The concentration of acetylcholine-related metabolites in salivary gland tissues and serum. (**e**–**h**) Impact of acetylcholine treatment on salivation in SAMP1/kl +/+ and SAMP1/kl -/- mice. (**i**–**k**) Acetylcholine injection induces the expression of several key enzymes involved in saliva secretion. Gene expression was measured by quantitative reverse transcription polymerase chain reaction (qRT-PCR). Data are presented as the mean ± standard deviation (SD). * p < 0.05, ** p < 0.01, *** p < 0.001. Data were compared by analysis of variance (ANOVA) with Bonferroni’s multiple comparisons test, in which each group was compared to the mean of the control.

To identify the Ach metabolite in SAMP1/kl mice, we examined Ach and Ach precursors. First, we analyzed the levels of acetyl-CoA, choline, and Ach in the salivary gland and serum of SAMP1/kl +/+ and SAMP1/kl -/- mice. As shown in Figure 2a–d, a reduction in Ach level was observed in the salivary gland and serum of SAMP1/kl -/- mice compared to those of SAMP1/kl +/+ mice, while acetyl-CoA and choline levels were slightly increased. Next, we evaluated the effects of Ach on saliva secretion in SAMP1/kl +/+ and SAMP1/kl -/- mice. Ach was injected intraperitoneally at a dose of 15 mg/kg. Consistent with previous studies, Ach induced salivation and increased serum Ca^2+^ levels in both SAMP1/kl +/+ and SAMP1/kl -/- mice (Figure 2e–h). The Ach-induced saliva secretion in SAMP1/kl -/- mice is significantly lower than that in SAMP1/kl +/+ mice, suggesting salivary gland dysfunction in these aging mice. Additionally, we also confirmed the Ach-induced upregulation of its receptors (M1AchR and M3 AchR) and calcium channel (ATP2a1), which participate in saliva secretion signaling (Figure 2i–k). Furthermore, treatment with Ach in human acinar cells (ACs) also induced the expression of cholinergic receptors (M1AchR and M3 AchR) and salivary gland functional markers (Supplementary Figure S2).

### 2.3. Choline Acetyltransferase (ChAT) is Downregulated in Primary Salivary Gland (PSGC) kl -/- Cells and SAMP1/kl -/- Mice

**Figure 3:**
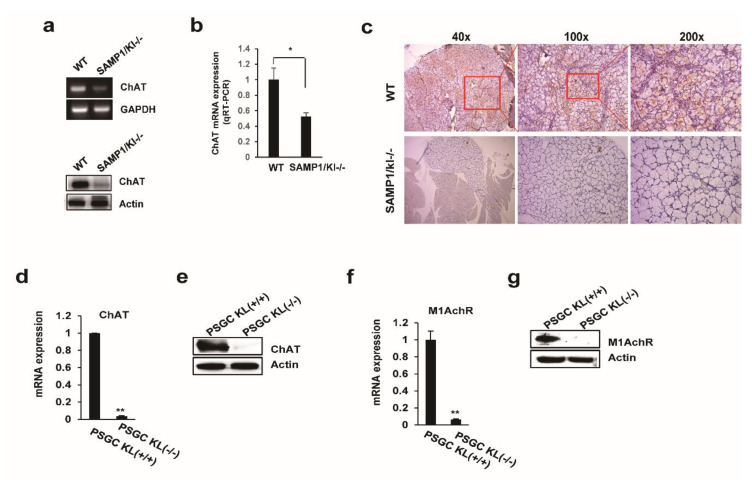
Downregulation of choline acetyltransferase (ChAT) in the Klotho-deficient model. (**a**,**b**) Comparison of ChAT expression in the salivary gland tissues of SAMP1/kl +/+ and SAMP1 kl -/- mice. Total RNA and total protein were extracted from the salivary gland tissues of SAMP1/kl +/+ and SAMP1 kl -/- mice, and ChAT expression was measured by RT-PCR, qRT-PCR, and Western blotting. (**c**) Immunohistochemistry staining of ChAT in 4 μm sections of salivary gland tissues from SAMP1/kl +/+ and SAMP1 kl -/- mice. (**d**–**g**) Comparison of cholinergic signaling related gene expression in primary salivary gland cells (PSGC) kl +/+ and PSGC kl -/- cell lines. Data are presented as the mean ± SD. * p < 0.05, ** p < 0.01.

ChAT catalyzes the transfer of an acetyl group from acetyl-CoA to choline, yielding Ach. To further understand the mechanism of the regulatory effect of Ach on saliva secretion, the expression of ChAT was measured using quantitative reverse transcription polymerase chain reaction (qRT-PCR), Western blotting, and immunohistochemistry. The RNA and protein levels of ChAT were reduced in the salivary gland tissues of SAMP1/kl -/- mice compared to those of SAMP1/kl+/+ mice (Figure 3a–c). Furthermore, we also measured the mRNA and protein expression levels of ChAT and M1AchR in primary salivary gland cells (PSGC kl +/+ and PSGC kl -/-). As shown in Figure 3d,e, we found that ChAT was significantly inhibited in PSGC kl -/- cells compared to PSGC kl -/- cells. Similarly, the expression of M1AchR was also reduced in SAMP1/kl -/- mice (Figure 3f,g). These results showed that the decrease in ChAT expression in SAMP1/kl -/- mice may impair the biosynthesis and cholinergic signaling of Ach.

### 2.4. ChAT Regulates the Expression of Salivary Gland Functional Genes

**Figure 4:**
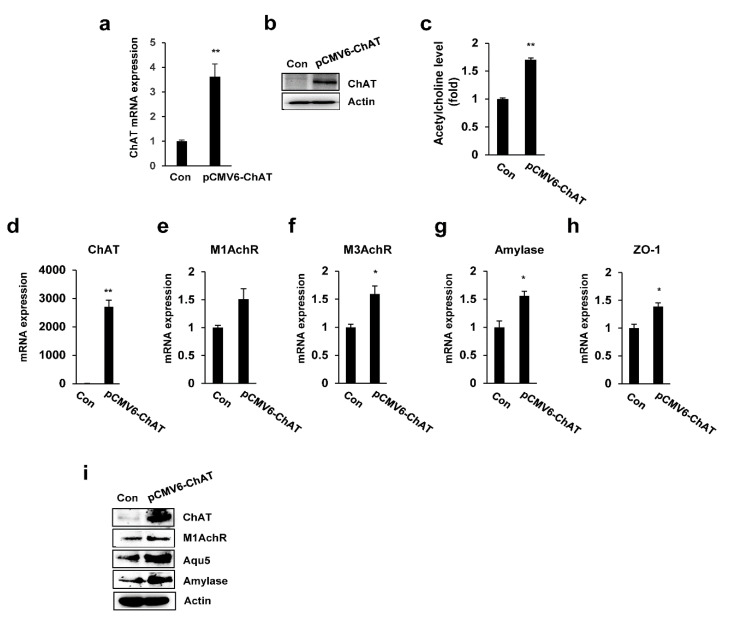
Overexpression of ChAT restored acetylcholine levels and improved salivary gland marker expression. (**a**–**c**) PSGC kl -/- were transfected with the ChAT expression plasmid (pCMV6-ChAT). After transfection, Western blotting and qRT-PCR were conducted to assess the ChAT expression level. The cellular acetylcholine concentration was also measured. (**d**–**i**) PSGC kl -/- cells were transfected with the ChAT expression plasmid (pCMV6-ChAT). After transfection, Western blotting and qRT-PCR were conducted to assess the ChAT and salivary gland marker expression levels. Data are presented as the mean ± SD. * p < 0.05, ** p < 0.01.

To directly assess the functional contribution of ChAT to salivary gland functional signaling, we investigated the changes in the expression of salivary gland functional-related genes upon the overexpression of ChAT in PSGC 7 kl -/- cells. We first evaluated the expression of ChAT in ChAT-overexpressing PSGC kl -/- cells by real-time quantitative RT-PCR and immunoblotting and observed abundant ChAR mRNA and protein expression (Figure 4a,b). In addition, ChAT overexpression significantly increased the Ach levels in PSGC kl -/- cells (Figure 4c). To confirm the functional activity of PSGC cells, we examined the expression of cholinergic receptors (M1AchR and M3 AchR) and salivary gland functional markers, such as α-amylase (AC secretion product) and ZO-1 (tight junction protein). qRT-PCR was used to validate the altered expression of these genes. ChAT-induced PSGC kl -/- cells significantly induced the expression of M1AchR, M3 AchR, α-amylase, and ZO-1 compared to vector-induced PSGC kl -/- cells (Figure 4d–g). Additionally, overexpression of ChAT also induced the protein expression of Ach receptors (M1AchR) and salivary gland functional markers (Amylase and Aqp5), as shown in Figure 4h.

### 2.5. Knockdown of ChAT Inhibits Salivary Gland Function

**Figure 5:**
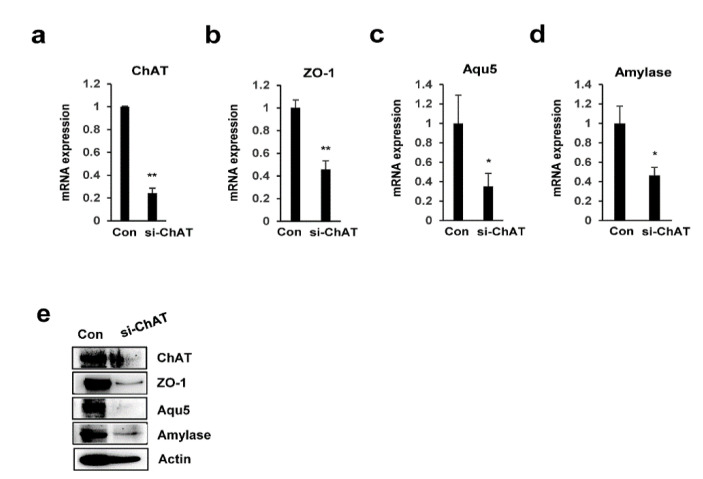
ChAT depletion diminished salivary gland functional marker expression. (**a**–**e**) PSGC kl +/+ cells were transfected with 1 nm of ChAT-specific siRNA (siChAT) for 48 h. Total mRNA and protein were extracted, and ChAT and salivary gland functional marker expression levels were evaluated by qRT-PCR and ChAT. Data are presented as the mean ± SD. * p < 0.05, ** p < 0.01.

To confirm the possible role of ChAT in the expression of salivary gland functional markers, we performed RNA interference to silence the ChAT gene. PSGC kl +/+ cells were transiently transfected with ChAT-siRNA or control-siRNA, a non-specific sequence control, for 48 h. As shown in Figure 5a, compared with control-siRNA, ChAT-siRNA significantly reduced ChAT mRNA levels after transfection. ChAT protein levels were also decreased in the cells transfected with ChAT-siRNA at 48 h. The mRNA expression of α-amylase, aquaporin-5, and ZO-1 was dramatically inhibited in ChAT-siRNA-transfected PSGC kl +/+ cells (Figure 5b–d). Consistent with mRNA expression results, the protein levels of α-amylase, aquaporin-5, and ZO-1 were also inhibited in PSGC kl +/+ cells transfected with ChAT-siRNA compared with those transfected with control-siRNA (Figure 5e).

### 2.6. In Vivo Salivary Gland Transduction Using Adeno-Associated Virus (AAV) Vectors Expressing ChAT

**Figure 6:**
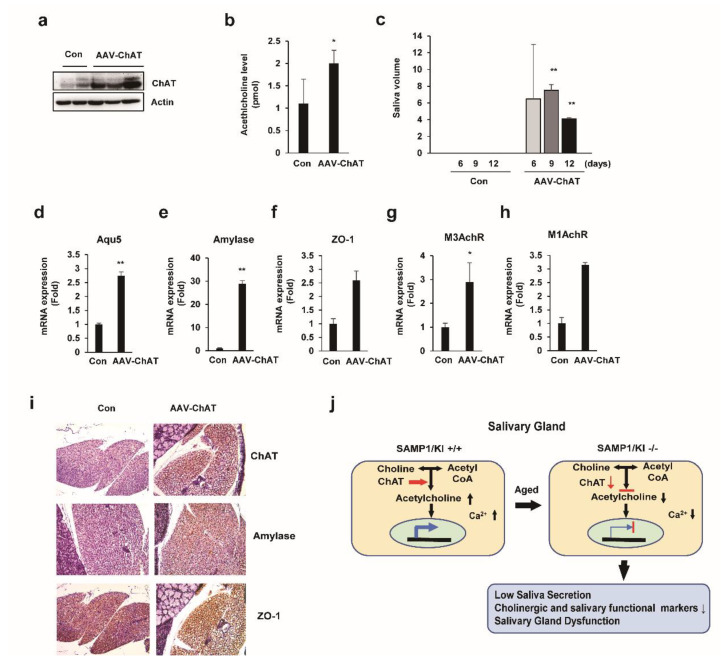
Regeneration of salivary gland function in SAMP1/kl -/- mice by adeno-associated virus (AAV)-ChAT delivery. SAMP1/kl -/- mice were either treated with phosphate-buffered saline (PBS, negative control) or AAV-ChAT at 6 weeks of age. Each mouse was injected with 2 × 10^10^ GCs of AAV-ChAT in 20 μL of PBS via intraglandular injection. Unstimulated saliva was collected every 3 days post injection. After 12 days, the mice were sacrificed, and the salivary glands were harvested for analysis. (**A**) Western blotting was conducted to confirm the expression of ChAT in the salivary glands. (**B**) The acetylcholine concentration was measured in the control and injected salivary glands. (**C**) Comparison of the secreted saliva volume between the control and injected group every 3 days. (**D**–**H**) Comparison of cholinergic signaling-related gene and functional marker expression in salivary gland tissues. (**I**) Immunohistochemistry analysis of the paraffin-embedded salivary glands derived from SAMP1/kl -/- and SAMP1/kl -/- mice injected with AAV-ChAT. (**J**) Summary of metabolic alterations in salivary gland of SAMP1/kl +/+ and SAMP1/kl -/- mice. Data are presented as the mean ± SD. * p < 0.05, ** p < 0.01.

To verify the functionality of adeno-associated virus (AAV)-ChAT vector in SAMP1/kl -/- mice, we transduced the AAV-ChAT to salivary glands of SAMP1/kl -/- mice. First, 2 × 10^10^ genome copies (GC) of AAV-ChAT was delivered to the salivary glands of SAMP1/kl -/- mice using intraglandular injection. The animals were euthanized after 12 days, the salivary glands were removed, and the ChAT protein levels examined to verify the transduction efficiency. The expression of ChAT was increased in AAV-ChAT injected SAMP1/kl -/- mice (Figure 6a). As expected, overexpression of ChAT significantly leads to an increase of acetylcholine levels in salivary gland of SAMP1/kl -/- mice (Figure 6b). To evaluate the effect of ChAT on the activity of salivary gland function, we measured saliva secretion. Importantly, in Figure 6c, we demonstrated that the amount of resting saliva is increased in AAV-ChAT injected SAMP1/kl -/- mice; at 9 and 12 days after AAV-ChAT injection, the rest of the saliva secretion increased by 70% and 40% of the control group, respectively, suggesting partial recovery of salivary gland function. There were no differences in the rest of the saliva secretion of the control group. Consistently, in vivo delivery of AAV-ChAT also induced the mRNA level of cholinergic receptors (M1AchR and M3AchR) and salivary gland functional markers (α-amylase, ZO-1, and Aqp5) in SAMP1/kl -/- mice (Figure 6d–h). Also, we confirmed the expression of α-amylase and Aqp5 in AAV-ChAT transduced human salivary gland cancer cells (HSGs) (data not shown). To further investigate the effects of AAV-ChAT on salivary gland function, we performed Immunohistochemistry analysis in AAV-ChAT injected SAMP1 kl -/- mice. As shown in Figure 6i, the expression of ChAT and salivary gland functional markers, α-amylase and ZO-1, strongly increased in the salivary gland of AAV-ChAT injected SAMP1 kl -/- mice compared to the control mice. These results demonstrate that ChAT partially restores the salivary gland function through regulating the biosynthesis and the cholinergic signaling pathway of acetylcholine, suggesting that ChAT may be a potential gene therapy for age-related salivary gland dysfunction. We summarize our findings about Ach metabolism in Figure 6j.

## 3. Discussion

Aging, a multifactor phenotype that is closely associated with a systemic reduction in body function, is also closely associated with metabolic alterations [22]. In this study, we identified the age-related metabolic and functional changes of salivary glands using an aging mouse (SAMP1/kl -/-) model. Our previous study showed that the levels of metabolites of antioxidants, such as GSH, were significantly decreased in SAMP1/kl -/- kidneys compared with SAMP1/kl +/+ kidneys [21]. Interestingly, in the salivary gland tissues of SAMP1/kl -/- mice, we not only found modifications similar to GSH metabolism in the kidney (Supplementary Figure S3) but also observed changes in Ach metabolism. Although the levels of the Ach precursors acetyl-CoA and choline did not change significantly, the levels of Ach were significantly reduced in the salivary glands of SAMP1/kl -/- mice compared to those of SAMP1/kl+/+ mice.

Ach is mainly synthesized by the enzyme ChAT, an enzyme that catalyzes the transfer of acetyl groups from acetyl-CoA to choline. Therefore, we hypothesized that the expression and Ach-producing activity of ChAT may be abnormal in SAMP1/kl -/- mice. In our study, we demonstrated that ChAT expression is importantly decreased in the salivary glands of SAMP1/kl -/- mice and PSGC kl -/- compared with the controls. Additionally, compared with SAMP1/kl +/+ mice, SAMP1/kl -/- mice exhibited significantly reduced expression of functional markers of salivary glands, including Amylase, Aqp5, and Zo-1. However, overexpression of ChAT could increase the levels of Ach and induce the expression of salivary gland markers in vitro and in vivo. On the other hand, inhibition of ChAT reduced the expression of salivary gland functional markers in PSGC kl +/+ cells. In vivo treatment with Ach also increased saliva secretion in SAMP1/kl -/- mice. These results suggested that the reduction in salivary gland function in aging may be linked with the attenuation of the ChAT/Ach mechanism.

Saliva secretion is initiated through the stimulation of parasympathetic and sympathetic branches of the autonomic nervous system. The glossopharyngeal nerve (CN IX), facial nerve (CN VII), and preganglionic nerves innervate salivary glands, and these nerves release several neurotransmitters, including Ach, to stimulate saliva secretion. During fluid and electrolyte secretion, Ach binds to muscarinic receptors, mainly M1 and M3, which activate phospholipase C to convert phosphatidylinositol bisphosphate to inositol triphosphate (IP3). IP3 triggers the release of Ca^2+^ from the endoplasmic reticulum, activates Ca^2+^-dependent apical Cl^-^ and basolateral K^+^ channels in the cell membranes and releases water [23]. Saliva played a fundamental role in the maintenance of oral health and, recently, it has been recognized as a potential candidate for non-invasive monitoring of heathy status. With the advantage of fast and easy to conduct, various methods were developed for early diagnosis of diseases, including oral diseases, neurodegenerative diseases, systemic infection, by evaluating the salivary biomarkers [24]. More specifically, periodontitis, the most prevalent oral disease, can be detected early by monitoring the level of soluble urokinase-type plasminogen activator receptor (suPAR), or Interleukin-6 (IL-6), or bacterial specific antibodies in saliva [25,26,27].

We observed that compared with SAMP1/kl +/+ mice, SAMP1/kl -/- mice have lower salivary volume and fewer cholinergic receptors (M1AchR and M3AchR) and calcium channels (ATP2a1), which participate in saliva secretion signaling. Importantly, treatment with Ach in SAMP1/kl -/- mice and PSGC kl -/- cells induced the expression of cholinergic receptors (M1AchR and M3AchR) and several key genes involved in saliva secretion in salivary glands. Additionally, Ach treatment increased Ca^2+^ levels and saliva secretion in both SAMP1/kl +/+ and SAMP1/kl -/- mice.

Recently, Park et al. successfully improved the cognitive function of aging mice by transplanting stem cells overexpressing ChAT to restore the depleted Ach level [28] Furthermore, activating Ach receptors with pharmaceutical agonists before irradiation reduces damage to salivary glands [29] Additionally, AAV-mediated Aquaporin 1 improved xerostomia [30] In our study, we observed that AAV-ChAT induced Ach levels in the salivary glands and serum, which in turn induced cholinergic receptor (M1AchR and M3 AchR) expression and improved saliva secretion. Taken together, our data demonstrated that ChAT/Ach levels may be a potential approach in the development of new therapies for salivary gland dysfunction.

As mentioned above, saliva secretion is controlled by the parasympathetic nervous system. Previously, it was reported that nerve growth factor (NGF) and nerve growth factor-induced protein C (NGFI-C or EGR4) can regulate the expression of ChAT transcriptionally, in both humans and mouse models [31,32]. In our previous study, we observed the downregulation of NGF and EGF in the salivary gland of aging mice; therefore, a signaling pathway between NGF/ChAT/Ach may exist. Further studies are needed to confirm this hypothesis.

In conclusion, our study demonstrated that accelerated aging SAMP1/kl -/- mice exhibit salivary gland dysfunction caused by metabolic alterations, including Ach metabolism. Compared with SAMP1/kl+/+ mice, SAMP1/kl -/- mice exhibited reduced saliva secretion, Ca^2+^ levels, cholinergic receptors, and several key genes involved in saliva secretion. This alteration is linked to a decrease in the expression of ChAT at both transcriptional and translational levels in SAMP1/kl -/- mice. Consistently, in vivo AAV-ChAT transduction improved the salivary gland function of SAMP1/kl -/- mice. The aging-related alteration in the expression of ChAT in SAMP1/Kl -/- mice might account for the concomitant changes in salivary gland functional activity. These results suggest that the ChAT/Ach pathway might have therapeutic benefit for the treatment of age-related salivary gland dysfunction.

## 4. Materials and Methods

### 4.1. Cell Culture and Reagents

The human submandibular gland (HSG) cell line and acini cell (AC) line were purchased from the Korea Cell Line Bank (KCLB, Seoul, Korea). Wild-type primary salivary gland cells (PSGC kl +/+) and mutant primary salivary gland cells (PSGC kl -/-) were generated as previously described [17]. The HSG, PSGC kl +/+ and PSGC kl -/- cell lines were cultured in Dulbecco’s modified eagle medium (DMEM) (Welgene Inc., Gyeongsanbuk-do, Korea) containing 10% fetal bovine serum (FBS), 100 units/mL penicillin, and 100 μg/mL streptomycin. The AC cell lines were cultured in keratinocyte serum-free medium (Keratinocyte SFM) (Thermo, Waltham, USA) containing 100 units/mL penicillin and 100 μg/mL streptomycin. All four cell lines were maintained in a 5% CO_2_ humidified atmosphere at 37 °C.

### 4.2. Metabolome Profile Analysis of Mouse Submandibular Salivary Gland Tissue by Capillary Electrophoresis Time-of-Flight Mass Spectrometry (CE-TOFMS) Analysis

Metabolome analyses were performed for 3 samples of mouse submandibular salivary gland tissue (wild type SAMP1/kl, 4-week-old SAMP1/kl -/-, and 8-week-old SAMP1/kl -/-) using CE-TOFMS in two modes for cationic and anionic metabolites. The samples were mixed with 50% acetonitrile in water (*v*/*v*) containing internal standards (20 μM for cation and 5 μM for anion measurement) and homogenized by a homogenizer (1500 rpm, 120 s × 4 times). The supernatant (400 μL × 2) was then filtered through a 5-kDa cut-off filter (ULTRAFREE-MC-PLHCC, Human Metabolome Technologies, Yamagata, Japan) to remove macromolecules.

### 4.3. Data Processing and Analysis

Peaks detected in the CE-TOFMS analysis were extracted using automatic integration software (Master Hands ver. 2.16.0.15 developed at Keio University) and used to obtain peak information, including *m*/*z*, migration time (MT), and relative peak area (peak area = metabolite peak area/(internal standard peak area × sample amount)). The following statistical analyses were performed: hierarchical cluster analysis (HCA) in Peak Stat ver. 3.18 (in-house software) and principal component analysis (PCA) in Sample Stat ver. 3.14 (in-house software). The profiles of peaks associated with putative metabolites were represented on metabolic pathway maps using Visualization and Analysis of Networks Containing Experimental Data (VANTED) 4 software. The abbreviations used for some metabolites shown in the pathway map are different from those used in the Human Metabolome Technologies (HMT) standard library. The pathway map was prepared based on known metabolic pathways according to the Kyoto Encyclopedia of Genes and Genomes (KEGG) database (http://www.genome.jp/kegg/) to exist in human cells

### 4.4. Biochemistry Analysis

The concentrations of Ach and choline were evaluated by a choline/Ach assay kit (Abcam, Cambridge, UK). The concentration of acetyl-CoA was evaluated by a Pico Probe™ Acetyl-CoA Fluorometric Assay Kit (Biovision, Milpitas, CA, USA). The concentration of Ca^2+^ was evaluated by the QuantiChrom™ Calcium Assay Kit (BioAssay Systems, Hayward, CA, USA). Experiments were conducted following the manufacturer’s instructions

### 4.5. Quantitative Reverse Transcription Polymerase Chain Reaction (qRT-PCR) Analysis

Total RNA was extracted from the transfected cells using TRIzol reagent (TaKaRa Bio Inc., Kusatsu, Japan) according to the manufacturer’s instructions. Quantitative reverse transcription PCR was performed using the GoTaq^®^ 1-Step RT-qPCR System kit (Promega, Madison, USA) according to the manufacturer’s protocol. The primer sets used are shown in Table 1. The relative expression level of target genes is represented by the 2-ΔΔCt value

### 4.6. Transfection Assay

The pCMV6-ChAT vector, which encodes the mouse-specific ChAT gene, was purchased from Origene (Rockville, United States). Plasmid transfection was performed using FuGENE^®^ 6 Transfection Reagent (Promega, Madison, WI, USA) according to the manufacturer’s protocol. siRNAs constructed for mouse ChAT were obtained from Bioneer (Bioneer, Deajeon, Korea). siRNA transfection was performed using Lipofectamine 2000 (Thermo, Waltham, MA, USA) following the manufacturer’s protocol. After 48 h, total RNA and protein were isolated and analyzed by qRT-PCR and Western blotting.

### 4.7. Western Blot Analysis

Cells were lysed using Radioimmunoprecipitation assay (RIPA) buffer (Biosesang, Seongnam, Korea) containing a protease inhibitor cocktail (1 μg/mL) and phosphatase inhibitor (1 μg/mL). Then, 15 μg of cell lysates were separated by 10% sodium dodecyl sulfate-polyacrylamide gel electrophoresis (SDS-PAGE) and transferred to a polyvinylidene difluoride (PVDF) membrane (Millipore, Burlington, NJ, USA). The membranes were blocked with 5% skim milk for 2 h and then incubated overnight at 4 °C with primary antibodies. ChAT antibody (sc-55557) was purchased from Santa Cruz Biotechnology (Santa Cruz, CA, USA). The tight junction protein Zonula occludens-1 (ZO-1) was detected with an anti-ZO-1 antibody (21773-1-AP) from Proteintech (Rosemont, IL, USA). The water channel protein Aqp5 was detected by an anti-aquaporin 5 antibody (AB15858) from Merck Millipore (Burlington, NJ, USA). Antibodies against β-actin (sc-47778) and α-amylase were purchased from Santa Cruz Biotechnology (Santa Cruz, CA, USA). The horseradish peroxidase (HRP)-conjugated secondary antibodies were purchased from Promega (Madison, WI, USA). The next day, the membranes were washed three times and then incubated with the corresponding secondary antibody for 1 h at room temperature. The protein signals were detected by a luminescence image analyzer (LAS-1000, Tokyo, Japan). The β-actin signal was used as a housekeeping standard.

### 4.8. Viral Vector Delivery and Immunohistochemistry Staining

SAMP1/kl -/- mice were generated as previously described [21]. A total of 6 SAMP1/kl -/- mice (4 weeks old, weighing 7–10 g) were used in the present study. The animals were housed in a controlled environment with a temperature of 21 ± 1 °C and a humidity of 50 ± 5%. Food and water were provided ad libitum. Adeno-associated virus overexpressing ChAT (AAV-ChAT) was purchased from Genecopoeia (Rockville, MA, USA). Through intraglandular injection, 2 × 10^10^ genome copies (GCs) were delivered to the salivary glands of SAMP1/Kl -/- mice. Six days after injection, the saliva secretion of all animals was examined every 3 days. After 12 days, the mice were sacrificed, and the salivary glands were excised, fixed in 10% buffered formalin and embedded in paraffin. Tissue sections (4-μm-thick) were stained using the avidinbiotin-peroxidase complex (ABC) immunohistochemical method. Endogenous peroxidase activity was blocked by 0.3% H_2_O_2_ in methanol for 10 min. Antigen retrieval was achieved by heating at 90 °C for 20 min in 10 mM citrate buffer (pH 6.0), followed by gradual cooling for 20 min. The same antibody used in the Western blot analysis was used to stain the target proteins. All animal procedures were performed under a protocol approved by the Chosun University Institutional Animal Care and Use Committee.

### 4.9. Statistical Analysis

All experiments were performed at least in triplicate. The results are expressed as the mean ± standard deviation (SD). The Student’s *t*-test and one-way analysis of variance (ANOVA) were used to determine the significant difference between the control and experimental groups. *P* values of less than 0.05 were considered statistically significant.

## Figures and Tables

**Table 1:** Primers Sequences.

Gene	Sequence
M1AchR	Forward: 5’– TCTCTGAATGCTGGAAGTAAAGA – 3’ Reverse: 5’– GAGACCCTAGATTCAGTCCCA – 3’
M3AchR	Forward: 5’– AGGGCTGACTACTTAATCTTGGATA – 3’ Reverse: 5’– TGCAAGGTCATTGTGACTCTC – 3’
ATP2α1	Forward: 5’– GAGCAGTTCGAAGACCTGCTTGTG – 3’ Reverse: 5’– CCTGTCAGGATGGACTGGTCGA – 3’
ChAT	Forward: 5’– GTTATAACCCCCAGCCTGAGGCC – 3’ Reverse: 5’– GGTCTCTCATGTCAACAAGGCTCGC – 3’
α-Amylase	Forward: 5’– GGTGCAACAATGTTGGTGTC – 3’ Reverse: 5’– ACTGCTTTGTCCAGCTTGAG – 3’
Zo-1	Forward: 5’– CGAGGCATCATCCCAAATAAGAAC – 3’ Reverse: 5’– TCCAGAAGTCTGCCCGATCAC – 3’
Aqp5	Forward: 5’– CGACCGTGTGGCTGTGGTCA – 3’ Reverse: 5’– GTGCCGGTCAGTGTGCCGTC – 3’
GAPDH	Forward: 5’– AGCCAAAAGGGTCATCATCTCTGC – 3’ Reverse: 5’– CCTTCCACAATGCCAAAGTTGTCA – 3’

## Data Availability

The datasets generated during and/or analyzed during the current study are available from the corresponding author on reasonable request.

## Supplementary Materials

The following are available online at https://www.mdpi.com/1422-0067/22/1/404/s1, Figure S1: Metabolites identified in principle metabolic pathways in SAMP1/kl +/+ and SAMP1/kl -/- salivary glands. Figure S2: Impact of in vitro acetylcholine treatment on the immortalized human acini cell line in a time-dependent manner. Figure S3: Klotho deficiency impairs glutathione metabolism in the salivary glands of SAMP1/kl -/- mice. Table S1: Changes in putative metabolites in the kidneys of SAMP1/kl+/+, SAMP1/kl -/-, and HL156A-treated SAMP1/kl -/- mice.
