# Supplementary material for: Choline Acetyltransferase Induces the Functional Regeneration of the Salivary Gland in Aging SAMP1/Kl -/- Mice
Source: Int J Mol Sci. 2021 Jan 2;22(1):404. doi: 10.3390/ijms22010404 (PMC7796039; doi:10.3390/ijms22010404)
Supplement: Supplementary file 1 [file ijms-22-00404-s001.zip › Suppl.Table 1. Changes in putative metabolites.docx]

| **HCA** |  |  |  |  |  |
| --- | --- | --- | --- | --- | --- |
| Line | HMT DB ^†^ | | Standardized Relative Area ^§^ | | |
|  | Compound name | KEGG ID | SAMP1/KL+/+ | SAMP1/KL-/- 1M | SAMP1/KL-/- 2M |
|  |  |  |  |  |  |
| 1 | Creatinine | [C00791](http://www.genome.jp/dbget-bin/www_bget?cpd:C00791) | -0.310 | -0.808 | 1.118 |
| 2 | 1-Pyrroline 5-carboxylic acid | [C03912](http://www.genome.jp/dbget-bin/www_bget?cpd:C03912) | -0.536 | -0.618 | 1.154 |
| 3 | 3-Phenylpropionic acid | [C05629](http://www.genome.jp/dbget-bin/www_bget?cpd:C05629) | -0.577 | -0.577 | 1.155 |
| 4 | 3-Phosphoglyceric acid | [C00197](http://www.genome.jp/dbget-bin/www_bget?cpd:C00197) | -0.577 | -0.577 | 1.155 |
| 5 | 3-Amino-2-piperidone |  | -0.577 | -0.577 | 1.155 |
| 6 | Urea | [C00086](http://www.genome.jp/dbget-bin/www_bget?cpd:C00086) | -0.728 | -0.412 | 1.140 |
| 7 | Choline | [C00114](http://www.genome.jp/dbget-bin/www_bget?cpd:C00114) | -0.737 | -0.401 | 1.138 |
| 8 | Adenosine | [C00212](http://www.genome.jp/dbget-bin/www_bget?cpd:C00212) | -0.749 | -0.386 | 1.136 |
| 9 | 2'-Deoxyguanosine | [C00330](http://www.genome.jp/dbget-bin/www_bget?cpd:C00330) | -0.858 | -0.240 | 1.098 |
| 10 | *O*-Acetylcarnitine | [C02571](http://www.genome.jp/dbget-bin/www_bget?cpd:C02571) | -0.873 | -0.218 | 1.091 |
| 11 | Glutathione (GSSG)_divalent | [C00127](http://www.genome.jp/dbget-bin/www_bget?cpd:C00127) | -0.898 | -0.179 | 1.078 |
| 12 | Thymidine | [C00214](http://www.genome.jp/dbget-bin/www_bget?cpd:C00214) | -0.902 | -0.174 | 1.075 |
| 13 | 3'-CMP 2'-CMP | [C05822 C03104](http://www.genome.jp/dbget-bin/www_bget?cpd:C05822) | -0.905 | -0.169 | 1.074 |
| 14 | 2'-Deoxycytidine | [C00881](http://www.genome.jp/dbget-bin/www_bget?cpd:C00881) | -0.914 | -0.154 | 1.068 |
| 15 | Betaine aldehyde_+H_2_O | [C00576](http://www.genome.jp/dbget-bin/www_bget?cpd:C00576) | -0.918 | -0.148 | 1.066 |
| 16 | cCMP 2',3'-cCMP | [C00941 C02354](http://www.genome.jp/dbget-bin/www_bget?cpd:C00941) | -0.927 | -0.132 | 1.060 |
| 17 | 1-Methyl-4-imidazoleacetic acid | [C05828](http://www.genome.jp/dbget-bin/www_bget?cpd:C05828) | -0.934 | -0.120 | 1.055 |
| 18 | Histamine | [C00388](http://www.genome.jp/dbget-bin/www_bget?cpd:C00388) | -0.963 | -0.071 | 1.033 |
| 19 | ATP | [C00002](http://www.genome.jp/dbget-bin/www_bget?cpd:C00002) | -0.974 | -0.051 | 1.024 |
| 20 | Citric acid | [C00158](http://www.genome.jp/dbget-bin/www_bget?cpd:C00158) | -0.975 | -0.048 | 1.023 |
| 21 | Guanosine | [C00387](http://www.genome.jp/dbget-bin/www_bget?cpd:C00387) | -0.995 | -0.009 | 1.005 |
| 22 | ADP | [C00008](http://www.genome.jp/dbget-bin/www_bget?cpd:C00008) | -1.005 | 0.011 | 0.995 |
| 23 | *S*-Adenosylhomocysteine | [C00021](http://www.genome.jp/dbget-bin/www_bget?cpd:C00021) | -1.052 | 0.115 | 0.938 |
| 24 | Inosine | [C00294](http://www.genome.jp/dbget-bin/www_bget?cpd:C00294) | -1.053 | 0.115 | 0.937 |
| 25 | dAMP | [C00360](http://www.genome.jp/dbget-bin/www_bget?cpd:C00360) | -1.058 | 0.129 | 0.929 |
| 26 | dCMP | [C00239](http://www.genome.jp/dbget-bin/www_bget?cpd:C00239) | -1.104 | 0.260 | 0.845 |
| 27 | Ribulose 1,5-diphosphate | [C01182](http://www.genome.jp/dbget-bin/www_bget?cpd:C01182) | -1.112 | 0.288 | 0.824 |
| 28 | γ-Butyrobetaine | [C01181](http://www.genome.jp/dbget-bin/www_bget?cpd:C01181) | -1.124 | 0.333 | 0.791 |
| 29 | Cysteinesulfinic acid | [C00606](http://www.genome.jp/dbget-bin/www_bget?cpd:C00606) | -1.126 | 0.342 | 0.784 |
| 30 | Ethanolamine | [C00189](http://www.genome.jp/dbget-bin/www_bget?cpd:C00189) | -1.129 | 0.356 | 0.773 |
| 31 | dTMP | [C00364](http://www.genome.jp/dbget-bin/www_bget?cpd:C00364) | -1.143 | 0.429 | 0.714 |
| 32 | UDP | [C00015](http://www.genome.jp/dbget-bin/www_bget?cpd:C00015) | -1.144 | 0.434 | 0.709 |
| 33 | Pipecolic acid | [C00408](http://www.genome.jp/dbget-bin/www_bget?cpd:C00408) | -1.146 | 0.447 | 0.698 |
| 34 | 2-Deoxyglucose 6-phosphate | [C06369](http://www.genome.jp/dbget-bin/www_bget?cpd:C06369) | -1.153 | 0.528 | 0.625 |
| 35 | NAD^+^ | [C00003](http://www.genome.jp/dbget-bin/www_bget?cpd:C00003) | -1.155 | 0.576 | 0.578 |
| 36 | 2-Hydroxyisobutyric acid |  | -1.155 | 0.593 | 0.562 |
| 37 | CMP-*N*-acetylneuraminate | [C00128](http://www.genome.jp/dbget-bin/www_bget?cpd:C00128) | -1.153 | 0.622 | 0.531 |
| 38 | XA0035 |  | -1.152 | 0.650 | 0.501 |
| 39 | Acetyl CoA_divalent | [C00024](http://www.genome.jp/dbget-bin/www_bget?cpd:C00024) | -1.149 | 0.671 | 0.478 |
| 40 | *myo*-Inositol 1-phosphate *myo*-Inositol 3-phosphate | [C01177 C04006](http://www.genome.jp/dbget-bin/www_bget?cpd:C01177) | -1.149 | 0.672 | 0.477 |
| 41 | 2-Hydroxyvaleric acid |  | -1.149 | 0.677 | 0.472 |
| 42 | Glyceraldehyde 3-phosphate | [C00118,C00661](http://www.genome.jp/dbget-bin/www_bget?cpd:C00118) | -1.147 | 0.692 | 0.454 |
| 43 | Glutaric acid | [C00489](http://www.genome.jp/dbget-bin/www_bget?cpd:C00489) | -1.143 | 0.714 | 0.429 |
| 44 | Pyridoxamine 5'-phosphate | [C00647](http://www.genome.jp/dbget-bin/www_bget?cpd:C00647) | -1.141 | 0.726 | 0.415 |
| 45 | Taurocholic acid | [C05122](http://www.genome.jp/dbget-bin/www_bget?cpd:C05122) | -1.134 | 0.756 | 0.377 |
| 46 | Ethanolamine phosphate | [C00346](http://www.genome.jp/dbget-bin/www_bget?cpd:C00346) | -1.126 | 0.786 | 0.340 |
| 47 | *N*-Acetylneuraminic acid | [C00270](http://www.genome.jp/dbget-bin/www_bget?cpd:C00270) | -1.114 | 0.821 | 0.293 |
| 48 | Diethanolamine | [C06772](http://www.genome.jp/dbget-bin/www_bget?cpd:C06772) | -1.081 | 0.891 | 0.190 |
| 49 | 5-Oxoproline | [C01879](http://www.genome.jp/dbget-bin/www_bget?cpd:C01879) | -1.059 | 0.928 | 0.131 |
| 50 | Cytidine | [C00475](http://www.genome.jp/dbget-bin/www_bget?cpd:C00475) | -1.057 | 0.930 | 0.127 |
| 51 | Pantothenic acid | [C00864](http://www.genome.jp/dbget-bin/www_bget?cpd:C00864) | -1.048 | 0.943 | 0.105 |
| 52 | Uric acid | [C00366](http://www.genome.jp/dbget-bin/www_bget?cpd:C00366) | -1.047 | 0.945 | 0.103 |
| 53 | Homoserine | [C00263](http://www.genome.jp/dbget-bin/www_bget?cpd:C00263) | -1.046 | 0.946 | 0.100 |
| 54 | CMP | [C00055](http://www.genome.jp/dbget-bin/www_bget?cpd:C00055) | -1.041 | 0.953 | 0.087 |
| 55 | Rhein | [C10401](http://www.genome.jp/dbget-bin/www_bget?cpd:C10401) | -0.979 | 1.020 | -0.042 |
| 56 | Carnitine | [C00318,C00487,C15025](http://www.genome.jp/dbget-bin/www_bget?cpd:C00318) | -0.971 | 1.027 | -0.056 |
| 57 | 1-Methylhistamine | [C05127](http://www.genome.jp/dbget-bin/www_bget?cpd:C05127) | -0.959 | 1.037 | -0.078 |
| 58 | Homocitrulline | [C02427](http://www.genome.jp/dbget-bin/www_bget?cpd:C02427) | -0.926 | 1.061 | -0.135 |
| 59 | Ribose 5-phosphate | [C00117](http://www.genome.jp/dbget-bin/www_bget?cpd:C00117) | -0.881 | 1.087 | -0.206 |
| 60 | Thiamine phosphate | [C01081](http://www.genome.jp/dbget-bin/www_bget?cpd:C01081) | -0.873 | 1.091 | -0.218 |
| 61 | *O*-Acetylhomoserine 2-Aminoadipic acid | [C01077 C00956](http://www.genome.jp/dbget-bin/www_bget?cpd:C01077) | -0.856 | 1.099 | -0.243 |
| 62 | *myo*-Inositol 2-phosphate |  | -0.820 | 1.114 | -0.294 |
| 63 | FAD_divalent | [C00016](http://www.genome.jp/dbget-bin/www_bget?cpd:C00016) | -0.771 | 1.130 | -0.359 |
| 64 | Dihydroxyacetone phosphate | [C00111](http://www.genome.jp/dbget-bin/www_bget?cpd:C00111) | -0.768 | 1.131 | -0.362 |
| 65 | Betaine | [C00719](http://www.genome.jp/dbget-bin/www_bget?cpd:C00719) | -0.761 | 1.133 | -0.372 |
| 66 | Asp | [C00049,C00402,C16433](http://www.genome.jp/dbget-bin/www_bget?cpd:C00049) | -0.729 | 1.140 | -0.411 |
| 67 | *N*^5^-Ethylglutamine | [C01047](http://www.genome.jp/dbget-bin/www_bget?cpd:C01047) | -0.724 | 1.141 | -0.417 |
| 68 | UDP-glucose UDP-galactose | [C00029 C00052](http://www.genome.jp/dbget-bin/www_bget?cpd:C00029) | -0.657 | 1.151 | -0.494 |
| 69 | Acetoacetamide | [C11106](http://www.genome.jp/dbget-bin/www_bget?cpd:C11106) | -0.647 | 1.152 | -0.505 |
| 70 | CoA_divalent | [C00010](http://www.genome.jp/dbget-bin/www_bget?cpd:C00010) | -0.639 | 1.152 | -0.514 |
| 71 | XC0016 |  | -0.577 | 1.155 | -0.577 |
| 72 | *S*-Lactoylglutathione | [C03451](http://www.genome.jp/dbget-bin/www_bget?cpd:C03451) | -0.577 | 1.155 | -0.577 |
| 73 | cAMP | [C00575](http://www.genome.jp/dbget-bin/www_bget?cpd:C00575) | -0.577 | 1.155 | -0.577 |
| 74 | Prostaglandin E_2_ | [C00584](http://www.genome.jp/dbget-bin/www_bget?cpd:C00584) | -0.577 | 1.155 | -0.577 |
| 75 | Octanoic acid | [C06423](http://www.genome.jp/dbget-bin/www_bget?cpd:C06423) | -0.577 | 1.155 | -0.577 |
| 76 | SDMA |  | -0.577 | 1.155 | -0.577 |
| 77 | Gly-Asp |  | -0.573 | 1.155 | -0.581 |
| 78 | *threo*-β-Methylaspartic acid | [C03618](http://www.genome.jp/dbget-bin/www_bget?cpd:C03618) | -0.568 | 1.155 | -0.587 |
| 79 | 1-Methylhistidine 3-Methylhistidine | [C01152](http://www.genome.jp/dbget-bin/www_bget?cpd:) | -0.549 | 1.154 | -0.605 |
| 80 | Adenylosuccinic acid | [C03794](http://www.genome.jp/dbget-bin/www_bget?cpd:C03794) | -0.506 | 1.152 | -0.646 |
| 81 | Trigonelline | [C01004](http://www.genome.jp/dbget-bin/www_bget?cpd:C01004) | -0.451 | 1.146 | -0.695 |
| 82 | Uridine | [C00299](http://www.genome.jp/dbget-bin/www_bget?cpd:C00299) | -0.447 | 1.146 | -0.698 |
| 83 | Pelargonic acid | [C01601](http://www.genome.jp/dbget-bin/www_bget?cpd:C01601) | -0.407 | 1.139 | -0.732 |
| 84 | UMP | [C00105](http://www.genome.jp/dbget-bin/www_bget?cpd:C00105) | -0.289 | 1.113 | -0.824 |
| 85 | Phosphorylcholine | [C00588](http://www.genome.jp/dbget-bin/www_bget?cpd:C00588) | -0.273 | 1.108 | -0.835 |
| 86 | Spermidine | [C00315](http://www.genome.jp/dbget-bin/www_bget?cpd:C00315) | -0.251 | 1.101 | -0.851 |
| 87 | UDP-N-acetylgalactosamine-2 UDP-N-acetylglucosamine-2 | [C00203 C00043](http://www.genome.jp/dbget-bin/www_bget?cpd:C00203) | -0.250 | 1.101 | -0.851 |
| 88 | *N*-Acetylglucosamine 6-phosphate | [C00357](http://www.genome.jp/dbget-bin/www_bget?cpd:C00357) | -0.234 | 1.096 | -0.862 |
| 89 | Butyrylcarnitine | [C02862](http://www.genome.jp/dbget-bin/www_bget?cpd:C02862) | -0.225 | 1.093 | -0.869 |
| 90 | *N*-Acetylglucosamine 1-phosphate | [C04256](http://www.genome.jp/dbget-bin/www_bget?cpd:C04256) | -0.180 | 1.078 | -0.898 |
| 91 | Fumaric acid | [C00122](http://www.genome.jp/dbget-bin/www_bget?cpd:C00122) | -0.113 | 1.052 | -0.938 |
| 92 | Nicotinamide | [C00153](http://www.genome.jp/dbget-bin/www_bget?cpd:C00153) | -0.101 | 1.047 | -0.946 |
| 93 | GDP-glucose GDP-mannose GDP-galactose | [C00394 C00096 C02280](http://www.genome.jp/dbget-bin/www_bget?cpd:C00394) | 0.022 | 0.989 | -1.011 |
| 94 | 2,3-Diphosphoglyceric acid | [C01159](http://www.genome.jp/dbget-bin/www_bget?cpd:C01159) | 0.071 | 0.963 | -1.034 |
| 95 | UDP-N-acetylgalactosamine-1 UDP-N-acetylglucosamine-1 | [C00203 C00043](http://www.genome.jp/dbget-bin/www_bget?cpd:C00203) | 0.230 | 0.865 | -1.095 |
| 96 | XA0065 |  | 0.286 | 0.826 | -1.112 |
| 97 | Hypoxanthine | [C00262](http://www.genome.jp/dbget-bin/www_bget?cpd:C00262) | 0.378 | 0.756 | -1.134 |
| 98 | Triethanolamine | [C06771](http://www.genome.jp/dbget-bin/www_bget?cpd:C06771) | 0.389 | 0.747 | -1.136 |
| 99 | Thiamine | [C00378](http://www.genome.jp/dbget-bin/www_bget?cpd:C00378) | 0.392 | 0.744 | -1.137 |
| 100 | *S*-Adenosylmethionine | [C00019](http://www.genome.jp/dbget-bin/www_bget?cpd:C00019) | 0.394 | 0.743 | -1.137 |
| 101 | Saccharopine | [C00449](http://www.genome.jp/dbget-bin/www_bget?cpd:C00449) | 0.413 | 0.727 | -1.140 |
| 102 | Putrescine | [C00134](http://www.genome.jp/dbget-bin/www_bget?cpd:C00134) | 0.473 | 0.676 | -1.149 |
| 103 | Ribulose 5-phosphate | [C00199,C01101](http://www.genome.jp/dbget-bin/www_bget?cpd:C00199) | 0.490 | 0.660 | -1.151 |
| 104 | XC0001 |  | 0.496 | 0.655 | -1.151 |
| 105 | Cysteine glutathione disulfide | [C05526](http://www.genome.jp/dbget-bin/www_bget?cpd:C05526) | 0.664 | 0.486 | -1.150 |
| 106 | UDP-glucuronic acid | [C00167](http://www.genome.jp/dbget-bin/www_bget?cpd:C00167) | 0.688 | 0.459 | -1.147 |
| 107 | Gln | [C00064,C00303,C00819](http://www.genome.jp/dbget-bin/www_bget?cpd:C00064) | 0.699 | 0.446 | -1.145 |
| 108 | GMP | [C00144](http://www.genome.jp/dbget-bin/www_bget?cpd:C00144) | 0.705 | 0.439 | -1.144 |
| 109 | Stachydrine | [C10172](http://www.genome.jp/dbget-bin/www_bget?cpd:C10172) | 0.712 | 0.431 | -1.143 |
| 110 | Malic acid | [C00149,C00497,C00711](http://www.genome.jp/dbget-bin/www_bget?cpd:C00149) | 0.716 | 0.427 | -1.143 |
| 111 | 5-Methyltetrahydrofolic acid | [C00440](http://www.genome.jp/dbget-bin/www_bget?cpd:C00440) | 0.750 | 0.385 | -1.135 |
| 112 | 3'-Dephospho CoA | [C00882](http://www.genome.jp/dbget-bin/www_bget?cpd:C00882) | 0.768 | 0.362 | -1.131 |
| 113 | Trp | [C00078,C00525,C00806](http://www.genome.jp/dbget-bin/www_bget?cpd:C00078) | 0.772 | 0.358 | -1.130 |
| 114 | ADP-ribose | [C00301](http://www.genome.jp/dbget-bin/www_bget?cpd:C00301) | 0.785 | 0.341 | -1.126 |
| 115 | CDP-choline | [C00307](http://www.genome.jp/dbget-bin/www_bget?cpd:C00307) | 0.787 | 0.338 | -1.125 |
| 116 | 3-Hydroxybutyric acid | [C01089,C03197](http://www.genome.jp/dbget-bin/www_bget?cpd:C01089) | 0.791 | 0.333 | -1.124 |
| 117 | 2-Aminoisobutyric acid 2-Aminobutyric acid | [C03665 C02261,C02356](http://www.genome.jp/dbget-bin/www_bget?cpd:C03665) | 0.801 | 0.319 | -1.121 |
| 118 | Aminoacetone | [C01888](http://www.genome.jp/dbget-bin/www_bget?cpd:C01888) | 0.812 | 0.305 | -1.117 |
| 119 | Asn | [C00152,C01905,C16438](http://www.genome.jp/dbget-bin/www_bget?cpd:C00152) | 0.819 | 0.296 | -1.115 |
| 120 | 2-Hydroxybutyric acid | [C05984](http://www.genome.jp/dbget-bin/www_bget?cpd:C05984) | 0.854 | 0.246 | -1.100 |
| 121 | Acetylcholine | [C01996](http://www.genome.jp/dbget-bin/www_bget?cpd:C01996) | 0.859 | 0.238 | -1.098 |
| 122 | Glucuronic acid Galacturonic acid | [C00191 C00333](http://www.genome.jp/dbget-bin/www_bget?cpd:C00191) | 0.860 | 0.237 | -1.097 |
| 123 | *N*-Acetylglucosylamine | [C01239](http://www.genome.jp/dbget-bin/www_bget?cpd:C01239) | 0.872 | 0.219 | -1.091 |
| 124 | Ala | [C00041,C00133,C01401](http://www.genome.jp/dbget-bin/www_bget?cpd:C00041) | 0.882 | 0.204 | -1.086 |
| 125 | ADMA | [C03626](http://www.genome.jp/dbget-bin/www_bget?cpd:C03626) | 0.889 | 0.193 | -1.082 |
| 126 | Lauric acid | [C02679](http://www.genome.jp/dbget-bin/www_bget?cpd:C02679) | 0.890 | 0.192 | -1.082 |
| 127 | Thr-Asp |  | 0.898 | 0.180 | -1.078 |
| 128 | Cystine | [C00491,C01420](http://www.genome.jp/dbget-bin/www_bget?cpd:C00491) | 0.898 | 0.179 | -1.078 |
| 129 | AMP | [C00020](http://www.genome.jp/dbget-bin/www_bget?cpd:C00020) | 0.907 | 0.165 | -1.072 |
| 130 | Fructose 6-phosphate | [C05345,C00085](http://www.genome.jp/dbget-bin/www_bget?cpd:C05345) | 0.907 | 0.165 | -1.072 |
| 131 | Xanthine | [C00385](http://www.genome.jp/dbget-bin/www_bget?cpd:C00385) | 0.914 | 0.154 | -1.068 |
| 132 | Glu-Glu | [C01425](http://www.genome.jp/dbget-bin/www_bget?cpd:C01425) | 0.926 | 0.134 | -1.060 |
| 133 | β-Ala | [C00099](http://www.genome.jp/dbget-bin/www_bget?cpd:C00099) | 0.940 | 0.111 | -1.051 |
| 134 | Fructose 1,6-diphosphate | [C00354](http://www.genome.jp/dbget-bin/www_bget?cpd:C00354) | 0.944 | 0.104 | -1.048 |
| 135 | Glucose 1-phosphate | [C00103](http://www.genome.jp/dbget-bin/www_bget?cpd:C00103) | 0.951 | 0.091 | -1.042 |
| 136 | *N*-Glycolylneuraminic acid | [C03410](http://www.genome.jp/dbget-bin/www_bget?cpd:C03410) | 0.968 | 0.060 | -1.029 |
| 137 | Lactic acid | [C00186,C00256,C01432](http://www.genome.jp/dbget-bin/www_bget?cpd:C00186) | 0.969 | 0.059 | -1.028 |
| 138 | ADP-glucose GDP-fucose | [C00498 C00325](http://www.genome.jp/dbget-bin/www_bget?cpd:C00498) | 0.971 | 0.055 | -1.026 |
| 139 | Glucose 6-phosphate | [C00668,C01172,C00092](http://www.genome.jp/dbget-bin/www_bget?cpd:C00668) | 0.975 | 0.047 | -1.023 |
| 140 | XC0132 |  | 0.981 | 0.036 | -1.018 |
| 141 | Ascorbate 2-glucoside | [C18339](http://www.genome.jp/dbget-bin/www_bget?cpd:C18339) | 0.987 | 0.026 | -1.013 |
| 142 | His | [C00135,C00768,C06419](http://www.genome.jp/dbget-bin/www_bget?cpd:C00135) | 0.988 | 0.023 | -1.011 |
| 143 | *N*^6^-Acetyllysine | [C02727](http://www.genome.jp/dbget-bin/www_bget?cpd:C02727) | 0.992 | 0.016 | -1.008 |
| 144 | *N*-Acetyllysine | [C12989](http://www.genome.jp/dbget-bin/www_bget?cpd:C12989) | 0.995 | 0.010 | -1.005 |
| 145 | *p*-Toluic acid *m*-Toluic acid *o*-Toluic acid | [C01454 C07211 C07215](http://www.genome.jp/dbget-bin/www_bget?cpd:C01454) | 0.998 | 0.004 | -1.002 |
| 146 | *N*-Acetylaspartic acid | [C01042](http://www.genome.jp/dbget-bin/www_bget?cpd:C01042) | 1.005 | -0.010 | -0.995 |
| 147 | Hydroxyproline | [C01157](http://www.genome.jp/dbget-bin/www_bget?cpd:C01157) | 1.014 | -0.028 | -0.986 |
| 148 | 3',5'-ADP | [C00054](http://www.genome.jp/dbget-bin/www_bget?cpd:C00054) | 1.022 | -0.046 | -0.976 |
| 149 | IMP | [C00130](http://www.genome.jp/dbget-bin/www_bget?cpd:C00130) | 1.024 | -0.049 | -0.975 |
| 150 | 2-Hydroxyglutaric acid | [C02630,C01087,C03196](http://www.genome.jp/dbget-bin/www_bget?cpd:C02630) | 1.025 | -0.053 | -0.972 |
| 151 | Ser | [C00065,C00716,C00740](http://www.genome.jp/dbget-bin/www_bget?cpd:C00065) | 1.028 | -0.058 | -0.970 |
| 152 | 5-Hydroxylysine | [C16741](http://www.genome.jp/dbget-bin/www_bget?cpd:C16741) | 1.029 | -0.061 | -0.968 |
| 153 | *N*-Acetylalanine |  | 1.043 | -0.091 | -0.951 |
| 154 | Theobromine | [C07480](http://www.genome.jp/dbget-bin/www_bget?cpd:C07480) | 1.049 | -0.107 | -0.942 |
| 155 | Glutathione (GSH) | [C00051](http://www.genome.jp/dbget-bin/www_bget?cpd:C00051) | 1.053 | -0.116 | -0.937 |
| 156 | Homocarnosine | [C00884](http://www.genome.jp/dbget-bin/www_bget?cpd:C00884) | 1.058 | -0.129 | -0.929 |
| 157 | Gly-Gly | [C02037](http://www.genome.jp/dbget-bin/www_bget?cpd:C02037) | 1.063 | -0.140 | -0.923 |
| 158 | *N*-Acetylmethionine | [C02712](http://www.genome.jp/dbget-bin/www_bget?cpd:C02712) | 1.064 | -0.145 | -0.920 |
| 159 | Isobutyric acid Butyric acid | [C02632 C00246](http://www.genome.jp/dbget-bin/www_bget?cpd:C02632) | 1.079 | -0.184 | -0.895 |
| 160 | Sedoheptulose 7-phosphate | [C05382](http://www.genome.jp/dbget-bin/www_bget?cpd:C05382) | 1.082 | -0.192 | -0.890 |
| 161 | Citrulline | [C00327](http://www.genome.jp/dbget-bin/www_bget?cpd:C00327) | 1.083 | -0.196 | -0.888 |
| 162 | γ-Glu-Cys | [C00669](http://www.genome.jp/dbget-bin/www_bget?cpd:C00669) | 1.084 | -0.198 | -0.886 |
| 163 | PRPP | [C00119](http://www.genome.jp/dbget-bin/www_bget?cpd:C00119) | 1.091 | -0.219 | -0.872 |
| 164 | *N*-Acetylornithine | [C00437](http://www.genome.jp/dbget-bin/www_bget?cpd:C00437) | 1.091 | -0.219 | -0.872 |
| 165 | 6-Phosphogluconic acid | [C00345](http://www.genome.jp/dbget-bin/www_bget?cpd:C00345) | 1.093 | -0.225 | -0.869 |
| 166 | Cys | [C00097,C00736,C00793](http://www.genome.jp/dbget-bin/www_bget?cpd:C00097) | 1.096 | -0.233 | -0.863 |
| 167 | Succinic acid | [C00042](http://www.genome.jp/dbget-bin/www_bget?cpd:C00042) | 1.098 | -0.238 | -0.859 |
| 168 | Uracil | [C00106](http://www.genome.jp/dbget-bin/www_bget?cpd:C00106) | 1.098 | -0.239 | -0.859 |
| 169 | Lys | [C00047,C00739,C16440](http://www.genome.jp/dbget-bin/www_bget?cpd:C00047) | 1.103 | -0.256 | -0.847 |
| 170 | Ophthalmic acid |  | 1.104 | -0.260 | -0.845 |
| 171 | Spermine | [C00750](http://www.genome.jp/dbget-bin/www_bget?cpd:C00750) | 1.104 | -0.261 | -0.844 |
| 172 | XC0061 |  | 1.113 | -0.291 | -0.822 |
| 173 | *N*-Acetylgalactosamine *N*-Acetylmannosamine *N*-Acetylglucosamine | [C01132 C00645 C00140](http://www.genome.jp/dbget-bin/www_bget?cpd:C01132) | 1.117 | -0.306 | -0.811 |
| 174 | Tyr | [C00082,C01536,C06420](http://www.genome.jp/dbget-bin/www_bget?cpd:C00082) | 1.119 | -0.311 | -0.807 |
| 175 | 4-Guanidinobutyric acid | [C01035](http://www.genome.jp/dbget-bin/www_bget?cpd:C01035) | 1.121 | -0.321 | -0.800 |
| 176 | Carnosine | [C00386](http://www.genome.jp/dbget-bin/www_bget?cpd:C00386) | 1.127 | -0.347 | -0.780 |
| 177 | Dyphylline | [C07819](http://www.genome.jp/dbget-bin/www_bget?cpd:C07819) | 1.128 | -0.350 | -0.778 |
| 178 | Argininosuccinic acid | [C03406](http://www.genome.jp/dbget-bin/www_bget?cpd:C03406) | 1.128 | -0.351 | -0.777 |
| 179 | Glycerophosphocholine | [C00670](http://www.genome.jp/dbget-bin/www_bget?cpd:C00670) | 1.128 | -0.352 | -0.776 |
| 180 | Met | [C00073,C00855,C01733](http://www.genome.jp/dbget-bin/www_bget?cpd:C00073) | 1.129 | -0.353 | -0.775 |
| 181 | Anserine_divalent | [C01262](http://www.genome.jp/dbget-bin/www_bget?cpd:C01262) | 1.130 | -0.358 | -0.772 |
| 182 | Leu | [C00123,C01570,C16439](http://www.genome.jp/dbget-bin/www_bget?cpd:C00123) | 1.131 | -0.365 | -0.766 |
| 183 | Daminozide Ala-Ala | [C10996 C00993](http://www.genome.jp/dbget-bin/www_bget?cpd:C10996) | 1.132 | -0.370 | -0.762 |
| 184 | Thr | [C00188,C00820](http://www.genome.jp/dbget-bin/www_bget?cpd:C00188) | 1.134 | -0.378 | -0.756 |
| 185 | Ornithine | [C00077,C00515,C01602](http://www.genome.jp/dbget-bin/www_bget?cpd:C00077) | 1.135 | -0.381 | -0.753 |
| 186 | Gly | [C00037](http://www.genome.jp/dbget-bin/www_bget?cpd:C00037) | 1.135 | -0.385 | -0.751 |
| 187 | *N*^6^,*N*^6^,*N*^6^-Trimethyllysine | [C03793](http://www.genome.jp/dbget-bin/www_bget?cpd:C03793) | 1.136 | -0.389 | -0.747 |
| 188 | Ascorbic acid | [C00072](http://www.genome.jp/dbget-bin/www_bget?cpd:C00072) | 1.142 | -0.423 | -0.719 |
| 189 | 1-Methylnicotinamide | [C02918](http://www.genome.jp/dbget-bin/www_bget?cpd:C02918) | 1.144 | -0.438 | -0.706 |
| 190 | Cys-Gly | [C01419](http://www.genome.jp/dbget-bin/www_bget?cpd:C01419) | 1.146 | -0.448 | -0.698 |
| 191 | Glu | [C00025,C00217,C00302](http://www.genome.jp/dbget-bin/www_bget?cpd:C00025) | 1.146 | -0.449 | -0.697 |
| 192 | Phe | [C00079,C02057,C02265](http://www.genome.jp/dbget-bin/www_bget?cpd:C00079) | 1.148 | -0.466 | -0.682 |
| 193 | Guanidoacetic acid | [C00581](http://www.genome.jp/dbget-bin/www_bget?cpd:C00581) | 1.150 | -0.484 | -0.666 |
| 194 | Ile | [C00407,C06418,C16434](http://www.genome.jp/dbget-bin/www_bget?cpd:C00407) | 1.151 | -0.499 | -0.652 |
| 195 | Arg-Glu |  | 1.152 | -0.508 | -0.644 |
| 196 | Arg | [C00062,C00792](http://www.genome.jp/dbget-bin/www_bget?cpd:C00062) | 1.155 | -0.561 | -0.594 |
| 197 | *N*,*N*-Dimethylglycine | [C01026](http://www.genome.jp/dbget-bin/www_bget?cpd:C01026) | 1.155 | -0.573 | -0.582 |
| 198 | Isopropanolamine | [C03194,C05771](http://www.genome.jp/dbget-bin/www_bget?cpd:C03194) | 1.155 | -0.577 | -0.577 |
| 199 | Ergothioneine | [C05570](http://www.genome.jp/dbget-bin/www_bget?cpd:C05570) | 1.155 | -0.577 | -0.577 |
| 200 | Isocitric acid | [C00311](http://www.genome.jp/dbget-bin/www_bget?cpd:C00311) | 1.155 | -0.577 | -0.577 |
| 201 | Xanthosine | [C01762](http://www.genome.jp/dbget-bin/www_bget?cpd:C01762) | 1.155 | -0.577 | -0.577 |
| 202 | Trimethylamine | [C00565](http://www.genome.jp/dbget-bin/www_bget?cpd:C00565) | 1.155 | -0.577 | -0.577 |
| 203 | Trimethylamine *N*-oxide | [C01104](http://www.genome.jp/dbget-bin/www_bget?cpd:C01104) | 1.154 | -0.603 | -0.552 |
| 204 | Pro | [C00148,C00763,C16435](http://www.genome.jp/dbget-bin/www_bget?cpd:C00148) | 1.154 | -0.619 | -0.535 |
| 205 | Val | [C00183,C06417,C16436](http://www.genome.jp/dbget-bin/www_bget?cpd:C00183) | 1.149 | -0.672 | -0.477 |
| 206 | Methionine sulfoxide | [C02989](http://www.genome.jp/dbget-bin/www_bget?cpd:C02989) | 1.148 | -0.681 | -0.467 |
| 207 | 5-Aminoimidazole-4-carboxamide ribotide | [C04677](http://www.genome.jp/dbget-bin/www_bget?cpd:C04677) | 1.142 | -0.717 | -0.425 |
| 208 | Hypotaurine | [C00519](http://www.genome.jp/dbget-bin/www_bget?cpd:C00519) | 1.137 | -0.744 | -0.392 |
| 209 | Terephthalic acid | [C06337](http://www.genome.jp/dbget-bin/www_bget?cpd:C06337) | 1.136 | -0.748 | -0.388 |
| 210 | Isovalerylcarnitine |  | 1.134 | -0.756 | -0.377 |
| 211 | *cis*-Aconitic acid | [C00417](http://www.genome.jp/dbget-bin/www_bget?cpd:C00417) | 1.131 | -0.768 | -0.363 |
| 212 | GDP | [C00035](http://www.genome.jp/dbget-bin/www_bget?cpd:C00035) | 1.129 | -0.773 | -0.356 |
| 213 | Tyr-Arg_divalent |  | 1.122 | -0.796 | -0.326 |
| 214 | Creatine | [C00300](http://www.genome.jp/dbget-bin/www_bget?cpd:C00300) | 1.118 | -0.810 | -0.308 |
| 215 | Myristoleic acid | [C08322](http://www.genome.jp/dbget-bin/www_bget?cpd:C08322) | 1.057 | -0.930 | -0.127 |
| 216 | Isobutyrylcarnitine |  | 1.047 | -0.946 | -0.101 |
| 217 | GABA | [C00334](http://www.genome.jp/dbget-bin/www_bget?cpd:C00334) | 1.004 | -0.996 | -0.008 |
| 218 | Gluconic acid | [C00257](http://www.genome.jp/dbget-bin/www_bget?cpd:C00257) | 0.981 | -1.018 | 0.038 |
| 219 | *N*-Acetylglutamic acid | [C00624](http://www.genome.jp/dbget-bin/www_bget?cpd:C00624) | 0.959 | -1.037 | 0.078 |
| 220 | Glycerol | [C00116](http://www.genome.jp/dbget-bin/www_bget?cpd:C00116) | 0.709 | -1.144 | 0.434 |
| 221 | XC0089 |  | 0.701 | -1.145 | 0.444 |
| 222 | 3-Indoxylsulfuric acid |  | 0.636 | -1.153 | 0.517 |
| 223 | Hexanoic acid | [C01585](http://www.genome.jp/dbget-bin/www_bget?cpd:C01585) | 0.623 | -1.153 | 0.531 |
| 224 | Isoglutamic acid | [C05574](http://www.genome.jp/dbget-bin/www_bget?cpd:C05574) | 0.573 | -1.155 | 0.582 |
| 225 | Glycerol 3-phosphate | [C00093](http://www.genome.jp/dbget-bin/www_bget?cpd:C00093) | 0.560 | -1.155 | 0.595 |
| 226 | GTP | [C00044](http://www.genome.jp/dbget-bin/www_bget?cpd:C00044) | 0.513 | -1.152 | 0.639 |
| 227 | Sarcosine | [C00213](http://www.genome.jp/dbget-bin/www_bget?cpd:C00213) | 0.491 | -1.151 | 0.659 |
| 228 | Serotonin | [C00780](http://www.genome.jp/dbget-bin/www_bget?cpd:C00780) | 0.402 | -1.138 | 0.737 |
| 229 | Carboxymethyllysine |  | 0.249 | -1.101 | 0.852 |
| 230 | Noradrenaline 6-Hydroxydopamine | [C00547](http://www.genome.jp/dbget-bin/www_bget?cpd:C00547) | 0.244 | -1.099 | 0.856 |
| 231 | NADP^+^ | [C00006](http://www.genome.jp/dbget-bin/www_bget?cpd:C00006) | 0.238 | -1.097 | 0.860 |
| 232 | Taurine | [C00245](http://www.genome.jp/dbget-bin/www_bget?cpd:C00245) | -0.008 | -0.996 | 1.004 |
