# Supplementary figures and images for: Choline Acetyltransferase Induces the Functional Regeneration of the Salivary Gland in Aging SAMP1/Kl -/- Mice
Source: Int J Mol Sci. 2021 Jan 2;22(1):404. doi: 10.3390/ijms22010404 (PMC7796039; doi:10.3390/ijms22010404)

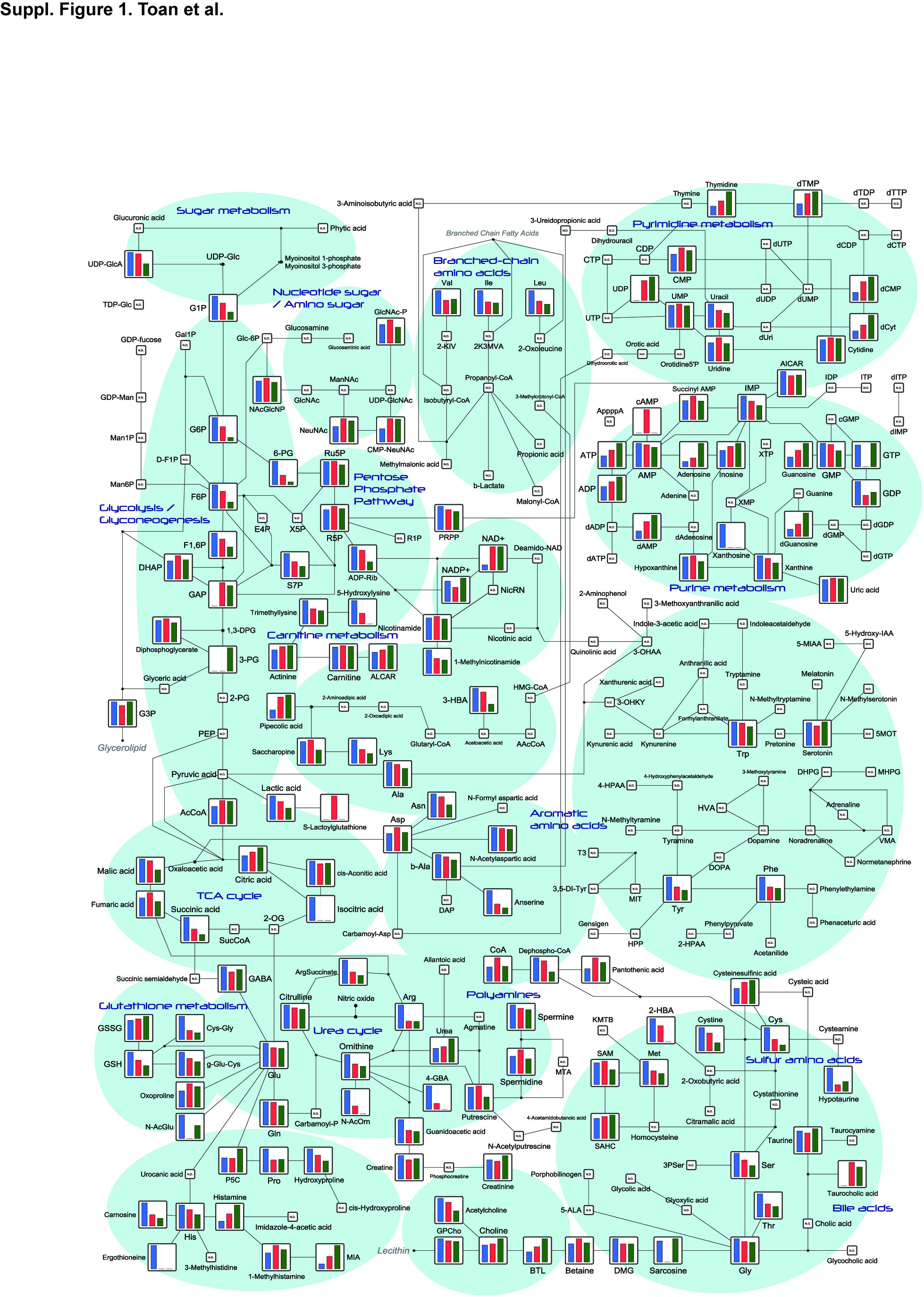

Supplement: Supplementary file 1 [file ijms-22-00404-s001.zip › Suppl. Figure 1.tif]

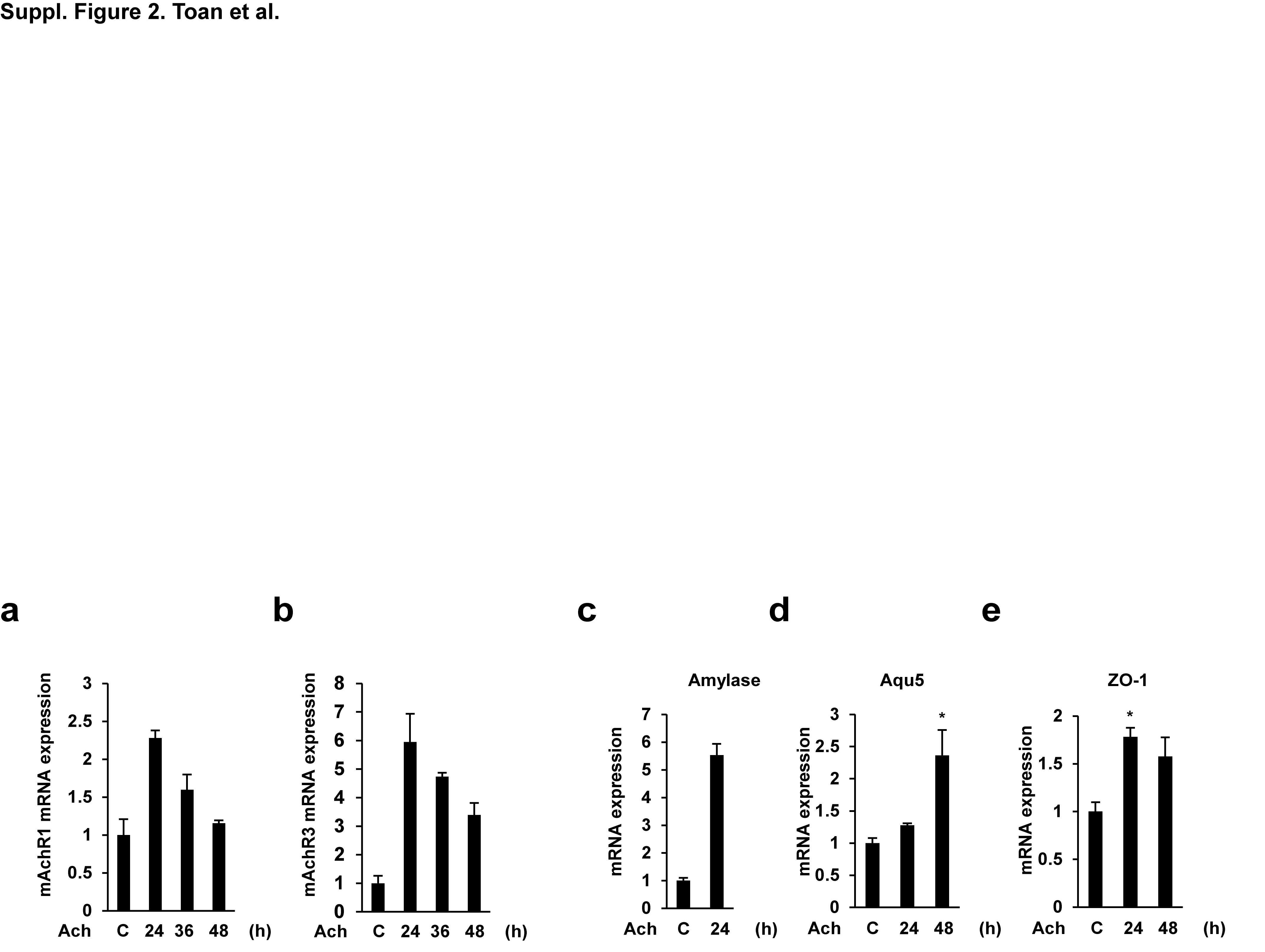

Supplement: Supplementary file 1 [file ijms-22-00404-s001.zip › Suppl. Figure 2.tif]

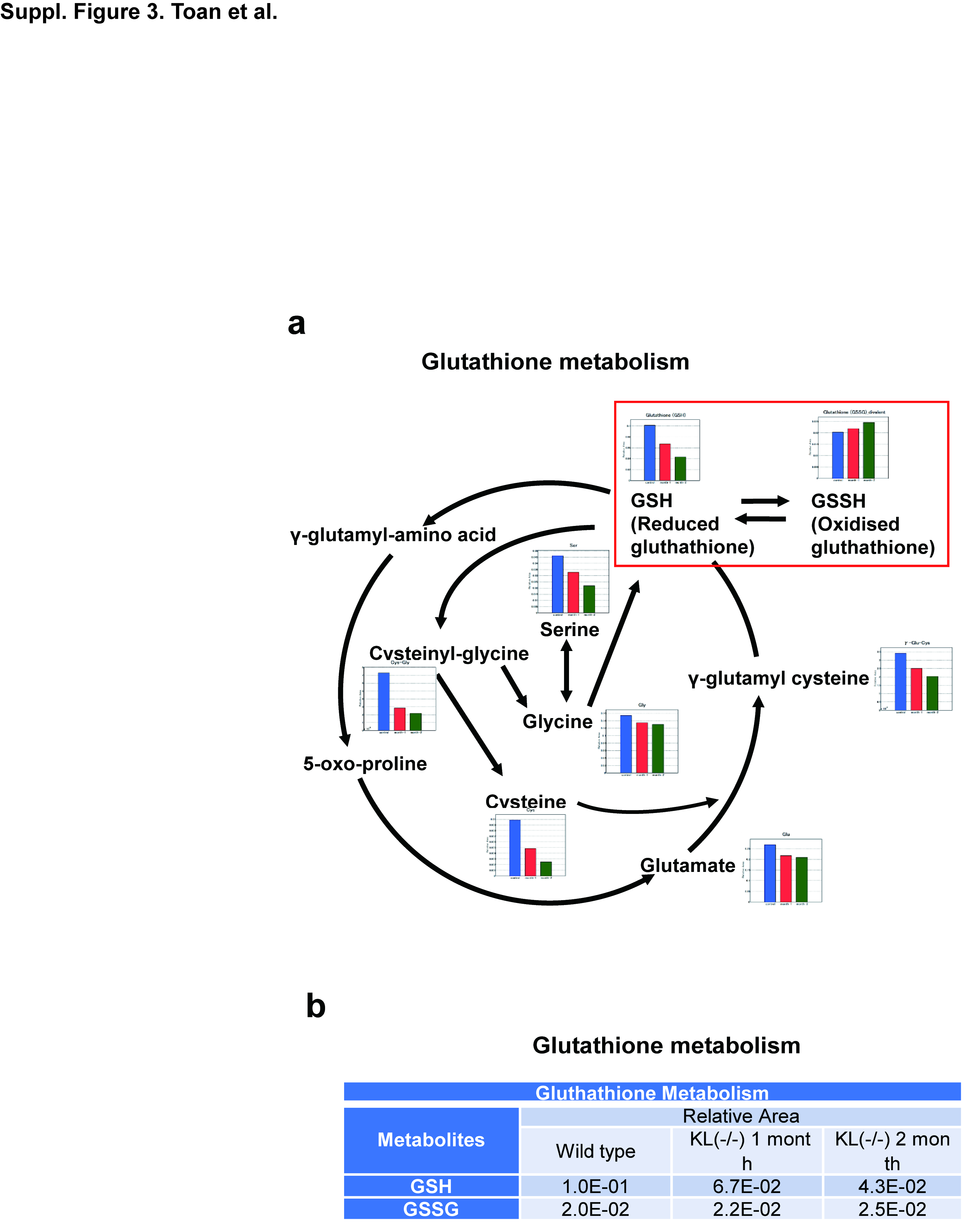

Supplement: Supplementary file 1 [file ijms-22-00404-s001.zip › Suppl. Figure 3.tif]
